# Multi-Variant TLS and SfM Photogrammetric Measurements Affected by Different Factors for Determining the Surface Shape of a Thin-Walled Dome

**DOI:** 10.3390/s20247095

**Published:** 2020-12-11

**Authors:** Grzegorz Lenda, Jakub Siwiec, Jacek Kudrys

**Affiliations:** Faculty of Mining Surveying and Environmental Engineering, AGH University of Science and Technology, 30-059 Kraków, Poland; jsiwiec@agh.edu.pl (J.S.); jkudrys@agh.edu.pl (J.K.)

**Keywords:** terrestrial laser scanning (TLS), structure from motion (SfM), accuracy analysis, dome, shell structures, deformation monitoring

## Abstract

The paper presents a combined analysis of terrestrial laser scanning (TLS) and structure from motion (SfM) photogrammetric measurement to determine an accurate model of the surface shape of a thin-walled dome. The analysis takes into account several factors that may affect the accuracy of measurement. In TLS measurements, these are related to scattering of the beam and its penetration into the structure of objects. Penetration of the beam into a synthetic structure changes the measured length. Shell moisture, caused by rainfall or dew effect, similarly affects the measured length, but the changes are dispersed. In the first case, it will change the size of the object, and in the second one, it generates measurement noise. SfM photogrammetric problems, such as object gloss and ambient reflection, lack of detail, and different results from software creating point clouds were also analyzed. An interesting observation was the significant influence of atmospheric pollution, sedimented on the lower half of the glossy dome, on increasing the accuracy of photogrammetric measurement. The analyses contain a number of cases that take into account the complex problems of obtaining and processing data of such facilities: periodic measurement, TLS and SfM photogrammetric measurement, measurement outside and inside the object, determination of the wall thickness, comparison with the project and free-sphere fitting, and use of dome rotation during TLS.

## 1. Introduction

Terrestrial laser scanning (TLS) is a useful tool used in deformation monitoring [1,2,3,4]. A comparison of TLS measurement results with precise leveling, tacheometric measurements and contact sensors confirms the possibility of using TLS for monitoring engineering objects at the level of several millimeters [2,3]. The approximation of a surface based on a point cloud provides accurate data on the shape of the surface to be tested. In laboratory conditions, it is possible to obtain accuracy of approximation of a steel, concrete, or wooden surface at the level of 1–3 mm [2,5,6,7].

However, the accuracy of scanning may decrease in the case of objects such as a measured dome. The laser beam can be scattered if the surfaces are moist or made of synthetic or glossy material. Moisture affects the measurements in different ways; it can decrease the reflection intensity, range, and accuracy [8]. In situations where the surfaces are non-absorbent, a layer of liquid appears on the object (in which the beam is scattered) due to precipitation or dew. Scanning results obtained for the measurement of synthetic materials are relatively little systematized. Different authors indicate deterioration of accuracy about values of a few to several dozen mm, depending on the material type [9,10]. In the paper [11], the influence of synthetics on scanning results was investigated and errors of up to several dozen mm were confirmed. These errors depend on the ability of the material to scatter the beam in its structure, conditioned by many factors. In case of measurements of glossy objects [12], the beam can be reflected between the object and the elements of the environment, causing the occurrence of errors of significant values.

The measured dome is a non-absorbent object, made of synthetic material and covered with a glossy varnish from the outside. These factors may, therefore, determine the accuracy of scanning, although the glossiness, due to the location of the dome above other elements, should not affect the results.

Ground-based photogrammetric techniques and photographs using an unmanned aerial vehicle (UAV) could be also used for measuring engineering objects. This technique is comparable to other ones, such as classic measurement and laser scanning [13,14,15], but the accuracy is lower. On the basis of photographs taken with the aid of a UAV, it is possible to obtain the accuracy of engineering or architectural objects usually of a few centimeters [16,17]. It should be noted that surface models created on the basis of UAVs may vary, depending on the type of surface, control targets, contrast, background lighting, reflections, shadows, etc. The accuracy of UAV-based photogrammetry may be comparable to the accuracy of laser scanning in good conditions and in perpendicular directions to the object being measured [18]. Photogrammetric methods are finding more and more applications in the precision industry [19,20,21,22] as non-contact measuring methods that ensure repeatability and accuracy and are non-invasive but not devoid of disadvantages. For example, the study [19] indicates the ability to quickly measure multiple points in short periods of time. A disadvantage of this technology is the need to use control targets for maintaining high accuracy. The authors consider structure from motion (SfM) methods to be of little use as stand-alone high-accuracy measurements, but they can be used as a basis for determining the precision measurement settings. In the paper [23], the authors describe the applied photogrammetric measurement system with the use of commercial off-the-shelf cameras in dynamic measurements inside a wind tunnel. The subjects of the research are smooth objects of the plane surface in order to examine their deformations in time. Due to the inability to control the background lighting in the tunnel and the lack of characteristic points on membranes and smooth metal surfaces, a dense grid of targets describing the object was used. One of the factors influencing the accuracy of photogrammetric measurements, especially in the case of objects with smooth or wet surfaces, was reflections. In the publication [24], the authors investigated the use of a polarization filter to reduce reflections on photographed objects in medical applications. The study concluded that a polarization filter can be used on a standard camera in structure from motion (bundle adjustment). It does not change the parameters of the lens in any significant way (it is balanced during the alignment), and thanks to the elimination of some reflections, it is possible to more accurately represent the tested surface. 

The measured dome is an object with a small number of characteristic control targets, especially from the outside. During photogrammetric measurement, there will be additional problems signaled by the aforementioned authors: reflections in the glossy varnish layer from the outside and difficulties with uniform backlight inside.

Planned measurements of the dome include a number of cases where problems related to accuracy of scanning and photogrammetric method may occur. The task of the multi-variant tests is to determine and compare them.

**Figure 1:**
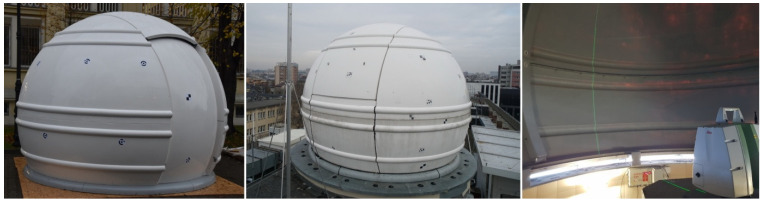
(**Left**) Dome after assembling; (**Center**) roof-mounted; (**Right**) internal view.

The research was aimed at conducting a combined analysis of measurement and computational methods to determine a model of the surface shape of a thin-walled dome. They contain a number of cases that take into account the complex problems of obtaining and processing data of such facilities. The dome of an astronomical observatory (Figure 1), with a diameter of 4 m, made of glass fiber, with a wall thickness of 6 mm, which was covered with lacquer of various properties from inside and outside, was measured. During the research, a number of factors influencing the accuracy of the analyses were taken into account. The measurement was carried out after the dome was assembled, and then, it was transported and mounted on the building to determine the potential deformations during these operations. Comparative measurements were made by means of terrestrial laser scanning and a photogrammetric method using a UAV platform. The internal and external sides of the shell were measured to compare the consistency of the results and to determine the wall thickness. The possibility of rotation of the dome was used during the scanning measurements, and the measurement was made from one scanners position. Scanning measurements were affected by factors that scatter the laser beam, resulting from moisture on the dome’s surface and penetration of the laser through the thin lacquer coating to the glass fiber layer. Photogrammetric UAV measurements were affected by environmental reflections on the smooth dome surface. The measured objects were subjected to analyses, comparing the results in the form of point clouds with the design model and the sphere model obtained from the free fitting. The point clouds from the photogrammetric method were determined in the structure from motion (SfM) software Agisoft and ContextCapture.

Due to the diversity of the research, the content is divided into chapters, dedicated to specific experiments, containing the description of the experiment, the results, and their analysis related to the results from previous experiments. The following tests were carried out:

- Laser scanning of the outer surface of the dome after assembling;
- Scanning of the outer surface of the dome after its transport and installation on the building;
- Comparative photogrammetric UAV measurement of the outer surface of the dome;
- Scanning of the internal surface of the dome;
- Comparative photogrammetric measurement of the internal surface of the dome.

## 2. Laser Scanning of the Outer Surface of the Dome after Assembling

**Figure 2:**
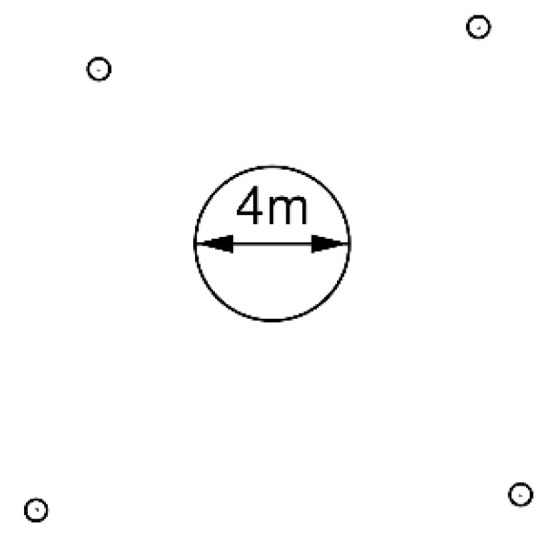
Scanner stations in relation to the dome.

The initial measurement determining the geometry of the dome once it had been assembled was taken with a Leica ScanStation C10 laser scanner with an accuracy of 6 mm and precision of 2 mm. Divergence of the laser beam was 0.1 mrad. The measurement was taken from four scanner stations located around the dome at a distance of 4–6 m from the object (Figure 2). The scanner locations’ heights were slightly above the middle stiffening extrusions of the dome. The connection of neighboring point cloud pairs was based on targets in the form of markers placed on the dome surface. Adjacent clouds overlapped by about 50%, but some of the targets were measured at unfavorable incident angles of the beam. Therefore, between each pair of scans, four of them were selected, which gave the smallest registration errors. In total, 16 targets were used (4 targets between every 4 pairs of scanner locations). The mean error of the point clouds’ registration in Leica Cyclone was 1 mm. The resultant cloud had an average resolution of 2 mm and it was similar for all subsequent experiments.

**Figure 3:**
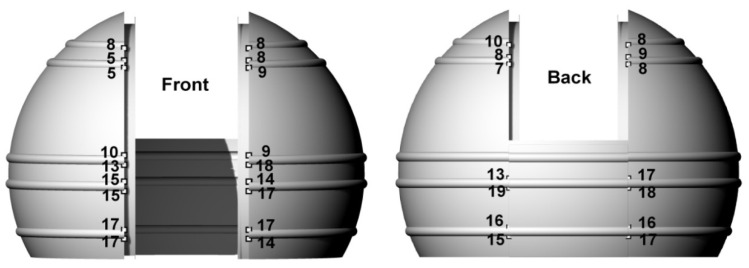
Deviations at points (mm) after a transformation using all the points.

The resulting cloud was compared with the solid design model. In order to make the comparison possible, it was necessary to transform the point cloud into a solid model coordinate system based on selected homological points. Transformation by applying specific translations and rotations by means of rigid body transformations was performed in Leica Cyclone. The only points that could be clearly distinguished in the cloud on the shell object were at the intersection of the edge of the slide gate and the horizontal stiffening extrusions, but due to local laser obscurations, not all of them could be used. The selected homological points are marked in Figure 3. As a result of the transformation, significant deviations were determined at some homological points. Deviations at the top of the dome were, on average, 8 mm, and at the bottom, 16 mm. Their values and locations indicate that the dome was deformed along the slide gate. Under the pressure of the upper fragments, larger deviations appeared in the lower part of the dome, which ceased to be a spherical object.

**Figure 4:**
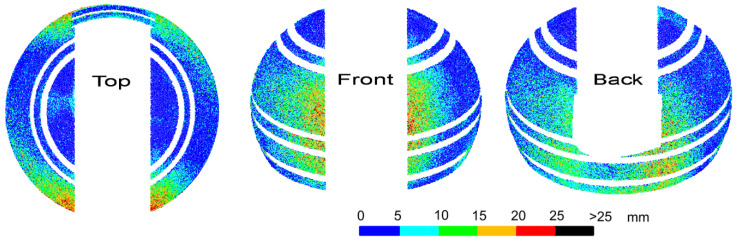
Laser scanning after the dome was assembled. Deviations of a point cloud from a free fitted sphere. The object was covered with rain drops.

Deformations of the object have a negative effect on the accuracy of matching the entire cloud to the design model. In addition, during the transformation of point clouds obtained from scanning and photos during subsequent experiments, there were problems in selecting the same homological points as in Figure 3. This was due to the lower position of the scanner in relation to the dome, which eliminated points in its upper part, and the low precision of point clouds obtained from UAV images. Thus, for each of the subsequent experiments, the cloud transformations to the design model coordinate system would be based on different homological points with different accuracy, and the comparison of the subsequent results would be distorted. However, since the analyzed object is a sphere, it was possible to perform free Least Squares Method (LSM) sphere fitting into the point cloud. Then, for each of the analyzed cases, deviations from the surface of the sphere were determined, allowing for direct comparison of the results. The values of deviations and their distribution are the basic criteria to determine the differences in the results of individual methods. Before the LSM method was applied, all elements not constituting the surface of the homogeneous sphere, i.e., horizontal stiffening extrusions and points located on a movable slider, had to be removed from the point clouds. The result of the fitting is shown in Figure 4, containing the deviations of points from the model sphere, grouped in intervals of 5 mm. Similar fits were made for all other experiments.

The deformations of the sphere that occurred at the homological points during the transformation are fully visible. They are located along the edge of the slider in the lower-central section. Larger deformations occur on the front side, which does not have a stiffener at the bottom of the dome. The deviation values are consistent with the transformation error values at the homological points and, at the top of the edge, have values up to 10 mm, and at the bottom—up to 20 or even 25 mm. However, deviations greater than 20 mm are distributed sporadically and randomly. They do not form continuous areas and do not correspond to the smooth surface of the dome. Furthermore, in other areas of the dome, random scattering of deviations of smaller values can be observed, which may suggest local, slight irregularities of the dome. In fact, the surface of the dome is smooth, but the random scattering of the results is due to the scattering of the laser beam when measuring a dome covered with small droplets of water after precipitation. In the article, two cases related to the scattering of the laser beam, which causes changes in the measured lengths, are examined. The first one is scattering on water drops, which randomly covered the dome and gave a random distribution of deviations of different values. The second, described in Chapter 6, is the refraction of light in the glass fiber layer, which changed length by a constant value. The authors conducted research related to changes in the measured length of the light scattering in surface structures (synthetic materials) [11]. Most synthetic materials cause changes in measured lengths and can be systematized. Drops of liquids, on the other hand, may have a different surface area, height, volume, and temperature, may cover different types of surfaces, and the light beam will fall at a different angle in each of them. Such measurements cannot be systematized because each result may be different. It is visible in the random dispersion of deviations over the entire surface of the object.

The average deviation of the sphere fit was 5.7 mm, and 0.075% of the points had deviations greater than 25 mm, which exceeds the values resulting from visible deformations of the dome along the slide gate. Deviations at the level of 25 mm were taken as a limit during further experiments. The radius of the fitted sphere was 1.996 m and is close to the design radius of 2.000 m.

To summarize, a comparison of the measurement results with the design model may encounter difficulties when the only homological points that can be distinguished on the dome are at the sites of the largest deformations of the object. Laser scanning combined with a free fitting of the sphere allowed for a proper evaluation of the deformations. However, the analysis was slightly disturbed by random deviations resulting from scanning the dome covered while with droplets of water. Moisture on the surface of non-absorbent objects adversely affects the precision of laser scanning.

## 3. Scanning of the Outer Surface of the Dome after Its Transport and Installation on the Building

After assembly, the dome was lifted by a crane to the roof of the building, where it was finally fixed. After about three months, another measurement was carried out using laser scanning to determine potential deformations resulting from these operations. The same instrument was used, i.e., Leica ScanStation C10. After being installed on the roof of the building, which has various installations, the positioning of the scanners’ set ups around the object was significantly restricted. Using the possibility of rotation of the dome, it was decided to measure the whole object from a single scanner’s position by rotating the dome in 90-degree intervals. The scanner was placed 3.5 m from the sphere’s surface, and its height was slightly below the middle stiffening ridge of the dome. On the dome, targets were placed so that each time the sphere was rotated by 90 degrees, eight targets were visible—four on the left and four on the right. This corresponded to the measurements from four scanner stations and registering neighboring clouds by the help of four targets, i.e., the earlier conditions of scanning after the dome was assembled. Measurement with the assumption of object rotation is a very good facilitation, but it is not certain whether the dome does not deform during rotation. If this were the case, there would be shifts between the point clouds registered for particular turning positions. They would also be visible when compared to the previous measurements from four positions with a stationary dome. Additionally, to check if the dome had not been deformed during the rotation, a single scan was performed from the inside of the dome. It will be presented in the further part of the study. The mean error of the point clouds registration in Leica Cyclone was 1 mm.

**Figure 5:**
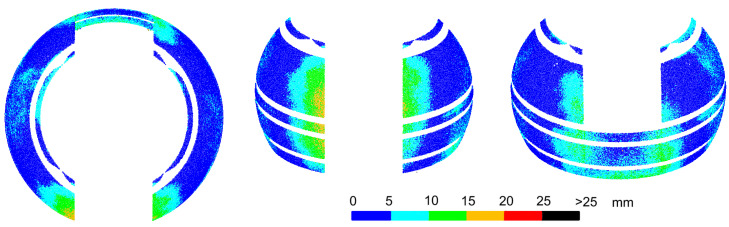
Laser scanning after transport and fixing of the dome. Deviations of a point cloud from a free fitted sphere. Dry object.

After the measurement, on the basis of experience from the previous point (comparing the cloud transformations to the design model and the free fitting), free fitting of the sphere into the point cloud was chosen. Next, the deviations of points from the sphere were determined in intervals of 5 mm. The results are shown in Figure 5.

The point cloud maintained consistency, and there were no visible, mutual shifts of quarters of the sphere which could occur if the dome were not rigid during rotation. The object had the same shape as when measured from four stations. The fragment of the upper surface of the dome was not measured because the scanner station was lower than the station from the previous experiment. The cloud had much less randomly scattered deviations, which is the result of the measurement of a dry object. Random deviations of values exceeding 25 mm occurred only for 0.002% of points, i.e., approximately 40-times less frequent than for an object covered with rain drops. The average sphere fitting deviation, which was 4.7 mm, was also smaller, indicating a general improvement in the precision of measurement for a dry object. Deformations of the sphere were distributed in the same places as for the dome after assembly, and they also had the same ranges of deviation values. This means that the transport and fixing of the dome did not cause any additional deformation. The radius of the determined sphere was 1.997 m and differed by 1 mm from the radius of the previous measurement.

In conclusion, the results of measuring the dry surface of the dome have noticeably better precision. The locations and values of the dome deformations from the previous measurement were confirmed. The object was not deformed during transport and fixing. Rotation of the dome, used during scanning from one station, also did not cause the dome to deform.

## 4. Comparative Photogrammetric UAV Measurement of the Outer Surface of the Dome

Significant difficulties in the location of observation stations were encountered when laser scanning the dome installed on the roof of the building, which influenced the decision to measure from one station while rotating the dome. However, it will not always be possible to find such a station. Additionally, lowering the scanner’s position in relation to the dome made it impossible to measure its upper part. The solution to this problem could be photogrammetric measurements from a UAV platform, on the basis of which it is also possible to create a point cloud. In order to analyze the accuracy of this method in comparison with laser scanning, a photogrammetric measurement was carried out as a manual flight of an unmanned aircraft with a non-metric camera. 

The measurements were carried out with DJI 900 unmanned aerial vehicle. A Sony ILCE-6000 camera was mounted on a 360-degree stabilized gimbal. The camera had a CMOS matrix of resolution 6000 × 4000 pixels, (with dimensions of 23.5 × 15.6 mm) and a lens of 15-mm focal length. The pixel size was 3.90 µm. As the mean distance to measured object was approx. 3.5 to 4 m, that resulted in acquiring the ground pixel of approx. 1 mm. A polarization filter was mounted on the lens to minimize reflections. The measurement as carried out as a manual flight with the pilot and camera operator. Flight paths were performed as horizontal passing-by at several heights around the measured dome. Horizontal and oblique photos were taken that covered the entire object, or with a minimum coverage of about 60% with a small or zero change in angle between the neighboring photographs. In total, 225 photographs were taken. In order to ensure the comparability of the UAV measurements with the scanning, the photographed markers placed on the surface of the sphere—black and white targets and automatic 12-bit markers of Agisoft Photoscan software—were used. These points were assigned coordinates measured on a point cloud obtained from the scanner and used in the study as constraints. 

Point clouds from photos were created using two programs with different calculation methodology: Agisoft Photoscan and Bentley ContextCapture. They belong to the group of Computer Vision programs based on the structure from motion technique used to create point clouds and 3D object models based on photos [25]. The general principle of their operation is the mutual orientation of photographs through the recognition of identical objects. Both programs first perform a search for similar objects in the pictures, based on local neighborhoods, using Scale-Invariant Feature Transform (SIFT)-based algorithms. Then, the mutual orientation of the pictures and their alignment in the local coordinate system is performed. In Agisoft Photoscan, the tie points (sparse cloud) were filtered to obtain certain connections, without using uncertain points (e.g., only found on a pair of photos). An important parameter is the required number of photos on which the same points are found. Photoscan relies on points found on a minimum pair of photos, whereas ContextCapture needs to find a point on at least three photos in order to be effectively used [26]. Thanks to the use of points with known coordinates which are identified on images, it is also possible to give the model a proper geo-reference and scale. Agisoft Photoscan builds a dense point cloud by finding additional similar points in the photos based on already-computed orientation of photos. Dense cloud filtering was deliberately not used in photogrammetry software. The use of filters at the dense matching stage meant that points with high reproduction uncertainty (most of the points on a smooth, shiny surface) were removed. Various degrees (powers) of filtration were tested, but due to the characteristics of the object, the algorithm removed a significant part of the observations. In order for the reader to be able to assess the appearance of the point cloud resulting from the reflections (top of the dome) compared to the places where there were no reflections (bottom of the dome), after generating the clouds, the points were not removed. The next step may be to create a mesh based on key points (a sparse cloud) or a dense point cloud. ContextCapture builds a mesh model directly from the resultant aerotriangulation cloud, the equivalent of a scarce point cloud from Photoscan, and can optionally generate a dense point cloud from it. However, the points are placed in a specific spatial resolution on the surface of the triangle grid. The main difference in the point clouds created in both programs is that the point cloud from ContextCapture only contains points located directly on the mesh, as a layer of zero “thickness”, while the point cloud from Photoscan is burdened with some noise, with some points created under or above the surface. For ContextCapture, this makes it difficult to detect model creation errors, especially on objects that have an irregular structure that cannot be described geometrically. 

**Figure 6:**
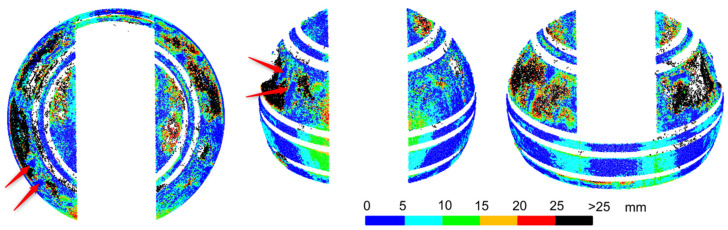
Comparative photogrammetric unmanned aerial vehicle (UAV) measurement. Deviations of the Agisoft Photoscan point cloud of the sphere from laser scanning (arrows indicate examples of target placement).

**Figure 7:**
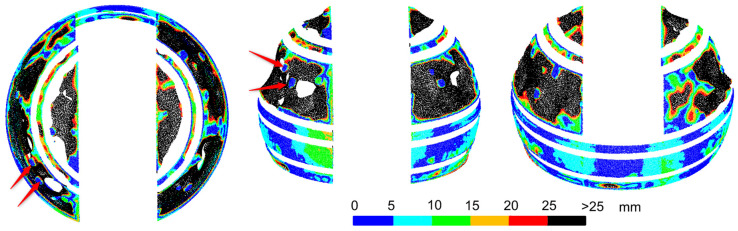
Comparative photogrammetric UAV measurement. Deviations of the ContextCapture point cloud from the sphere from laser scanning (arrows indicate examples of target placement).

The results obtained for both programs were significantly different from those obtained with a laser scanner. For Agisoft Photoscan, the radius of the freely fitted sphere was 1.966 m, and for ContextCapture, 1.972 m. In both cases, it differed by about 30 mm from the designed radius. It was decided to compare the point clouds to the sphere determined from laser scanning, whose radius differed only by 3 mm from the designed one. Deviations from the sphere for the Agisoft Photoscan point cloud are shown in Figure 6, and from ContextCapture, in Figure 7. As the control points used for scaling the photogrammetric point cloud originated from the TLS measurement, the problem of error propagation arose. In the upper part of the dome, small, regular areas of deviations with the smallest values, i.e., up to 5 mm, were visible (partially in Figure 6, especially in Figure 7). These are the places where the control points were glued. At these points, the UAV and TLS clouds differed from the sphere by no more than 5 mm. The influence of scanning accuracy on the photogrammetric measurements was, therefore, within this range, although it was not strictly determined. The point cloud obtained in Agisoft Photoscan had a high noise level. Some points were located at a distance of several dozen centimeters from the sphere surface. In Figure 6, they are partially covered by points with smaller deviations. The average deviation from the sphere was 44 mm. Overall, 30% of the points had deviations greater than 25 mm, while for scanning measurements, such deviations were very few (0.075% for the wet dome and 0.002% for the dry dome). In terms of overall statistics, similar results were obtained for the cloud from ContextCapture. The average deviation was smaller and reached 35 mm, while more deviations—as many as 36%—exceeded 25 mm. A comparison of Figure 6 and Figure 7 shows that the results of the two programs differ substantially. As mentioned above, the point cloud from Agisoft Photoscan has significant noise, but after filtering out the points with large deviations, obscured by points with smaller deviations, there remains a relatively significant surface area whose deviations are within 25 mm. The cloud from ContextCapture has much less noise; the points do not obscure each other, but after the removal of deviations of more than 25 mm, very little observation remains for the upper part of the dome. None of the results are comparable to laser scanning and are not suitable for assessing the shape of an object, but Agisoft Photoscan provides better results.

**Figure 8:**
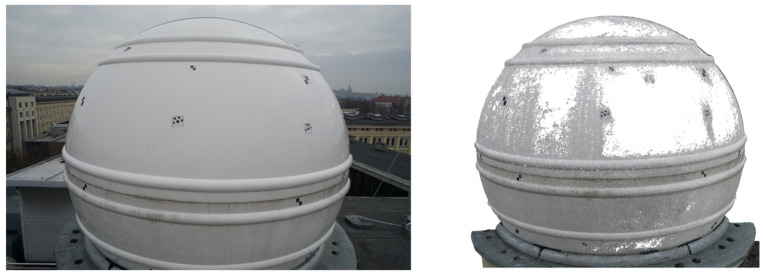
Comparison of the regions with pollutants and without. A photo of the dome (**left**) and the point cloud generated from Photoscan (**right**). To make the drawing easier to read, points with deviations over 25 mm have been removed from the cloud.

The comparison of the deviations on the upper and lower parts of the dome appears interesting. At the top, rainfall washes away pollutants, leaving the surface shiny and reflecting environmental elements that affect the captured image. In both programs, large deviations occurred at this part of the dome. The pollutants washed away from the top accumulated on the bottom part, making it more matte, which prevented reflections (Figure 8).

The lower part of the dome from both programs has deviations of much smaller values. The dirt on the surface was the only noticeable and distinct factor that differentiated the top and bottom of the dome, causing the bottom to be matte, without reflections and with visible streaks, and the top to shine. The matte spots gave a smaller distortion of the sphere and the regions with a glossy surface were covered with a bigger distortion or lack of measurement. It is an interpretation supported by research from other authors showing problems with reflections [24,27]. For Agisoft Photoscan, larger differences from the scanner occurred only in a narrow strip in the lower, central part of the back of the dome. For ContextCapture, the deformations of the object were also in the same places and with similar values as observed by the scanner. However, apart from these, there were also deviations of the same size at different points in the lower part of the dome. Without comparison with scanning results, it would not be possible to recognize which of them are the real deformations of an object and which are the result of errors in cloud modeling by the software. 

In summary, the quality of the models built from photos is very much dependent on the texture and glossiness of the dome. In places where the dome was glossy and reflected environmental elements, the accuracy of cloud generation in both programs was low and did not allow for analysis of the actual shape of the surface. The matte surface with streaks enabled obtaining an accuracy similar to laser scanning, but only in Agisoft Photoscan, which resulted from the algorithm used to generate point clouds.

## 5. Scanning of the Internal Surface of the Dome

**Figure 9:**
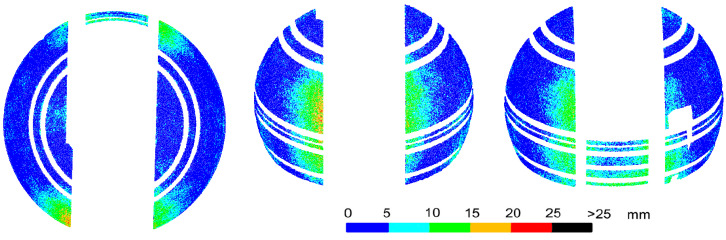
Laser scanning of the dome interior. Deviations of a point cloud from a free fitted sphere.

Previous experiments have identified a number of concerns and problems: uncertainty about the stiffness of the dome during measurements that used rotation of the object, inability to scan the upper part of the dome from the outside, and the fact that it is possible to obtain a full point cloud from UAV images, but with low accuracy due to the gloss of the surface. Due to the fact that the dome has a thin wall with a design thickness of 6 mm, an additional laser scan was carried out from the inside of the dome to obtain a full point cloud for the entire object. It was performed to finally verify the consistency of the point cloud obtained from scanning the outside of the dome because the measurement of the entire sphere could be made from one station located in the center of the dome. Based on internal and external scanning, the possibility of correct determination of the dome wall thickness was also evaluated. The same instrument was used, i.e., Leica ScanStation C10. After removing the elements which did not constitute a homogeneous sphere (gate, stiffening extrusions, and internal elements of the installation), the sphere was fitted with the LSM method. The deviations from the sphere were then determined and are shown in Figure 9.

**Figure 10:**
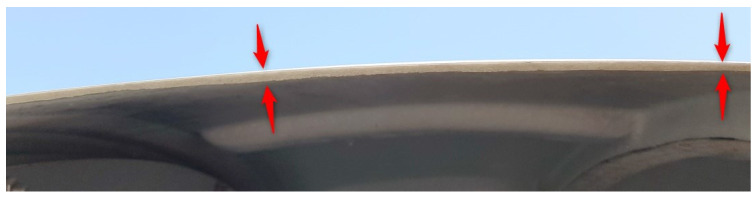
Unevenness of the internal surface, affecting the thickness of the dome wall.

The locations and values of the deviations correspond to the results for measuring the external surface of the dome. The outer side had a smooth surface, while the inner side, made by the boatbuilding method, had unevenly distributed surface roughness with an amplitude estimated at about 2–3 mm. To some extent, they may have affected small differences in deviation between the internal and external surfaces. The average deviation from the sphere was 4.3 mm (4.7 mm for the external surface). For both surfaces, deviations exceeding 25 mm were at the same level of 0.002%. The determined radius of the sphere was 1.994 m, which means that with an external sphere radius of 1.997 m, the wall thickness is only 3 mm, whereas the design is 6 mm. Most synthetic materials, including the glass fiber, of which the dome was made, scatter the laser beam within their structure, increasing the length measurement result. Thus, the following experiment was carried out: a scan of a fragment of the external and internal sides of the dome was performed, and then, the measurements were repeated after sticking a sheet of paper tightly adjacent to the dome in the same place. The sheet of paper prevented the laser from penetrating the synthetic material. Then, the distance between the covered cloud and the uncovered one was compared. The difference for the external surface was 0.3 mm, taking into account the thickness of the sheet, which can be considered negligible. The difference for the internal surface was 2.1 mm. The external surface was covered with a thick lacquer coating, effectively protecting against penetration of the laser beam into the fiberglass. On the internal surface, however, a thin, transparent layer of lacquer was applied, which did not sufficiently protect against the penetration of the laser into the structure of the material. This increased the measured length by a determined value of about 2 mm. Taking into account such a correction, the wall thickness was 5 mm. In addition, taking into account the above-mentioned unevenness of the inner surface, as shown in Figure 10, it can be estimated that the value of 5 mm corresponds to the actual average wall thickness of the dome.

In summary, the scan of the internal side of the dome positively verified the consistency of the point cloud for scanning the outer surface involving the dome rotation. The object has the same shape as when scanned from the outside. There was a problem with the penetration of the laser beam into the structure of the material, which should be taken into account when measuring synthetic materials. After determining the appropriate correction, the difference in wall thickness between the design and measurement values was 1 mm.

## 6. Comparative Photogrammetric Measurement of the Internal Surface of the Dome

Photogrammetric measurements of the external part of the dome were affected by large errors caused by reflections of the surrounding objects on the smooth surface covered with glossy lacquer. In places where the surface was matted by pollution, the errors were significantly reduced. The inner side of the dome had an uneven texture and was covered with matte lacquer. This makes it possible to obtain results of good accuracy, provided that the interior of the object is adequately illuminated. The photogrammetric measurement of the inner surface of the dome was taken as a series of ground photographs using a Nikon D5500 digital SLR camera with resolution of 6000 × 4000 and with a focal length of 18 mm. The matrix of sensors was 23.5 × 15.6 mm, with a pixel size of 3.9 µm. As the photos were taken from opposite walls of the dome, the average distance to the photographed surface was approximately 3.5 m. That resulted in acquiring approximately 0.8 mm ground pixel. In order to eliminate the effect of grain size, a third measurement was performed under identical conditions, assuming a constant ISO sensitivity of 400. This significantly reduced the grain size of the images without causing a significant decrease in edge sharpness. Measurements were taken as series of three parallel, vertical stripes of photos, with a mutual coverage of at least 80%. In total, 383 photos were used in further processing. Natural control points on the inner surface of the dome (characteristic points of markings and screws that are easy to recognize in photographs and scans) were used as homological points to the scanner’s point cloud coordinate system.

**Figure 11:**
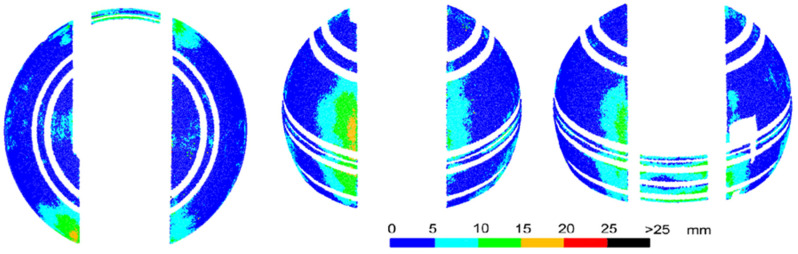
Comparative photogrammetric measurement of the dome interior. Deviations of the point cloud from the Agisoft Photoscan structure from motion (SfM) from a sphere freely fitted into the point cloud.

**Figure 12:**
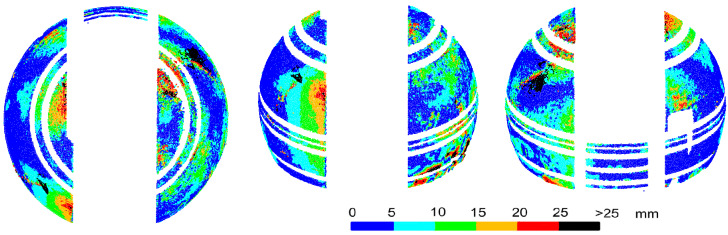
Comparative photogrammetric measurement of the dome interior. Deviations of the point cloud from the ContextCapture SfM from a sphere freely fitted into the point cloud.

After creating point clouds in Agisoft Photoscan and ContextCapture, the spheres were fitted into point clouds that had a radius of 1.991 m in both cases. Taking into account the sphere radius of the external scanning (1.997 m), the wall thickness of the external and internal dome was 6 mm, which was the same as the design. Taking Figure 9 into consideration, it can be estimated that the average wall thickness may have been slightly smaller than the design one. However, the internal radius from photogrammetric measurements was closer to the design than the radius from scanning inside the object, without taking into account any reduction in the laser penetration of the dome material. For external photogrammetric measurements, the values of the spherical radius were significantly different from the real radius, so the cloud deviations were related to the sphere determined from the scanning. For internal photogrammetric measurements, the sphere radius values were similar to the TLS results, and the deviations of the point clouds could be determined with respect to these spheres. The deviations of the point cloud from Agisoft Photoscan from the fitted sphere are shown in Figure 11, and from ContextCapture, in Figure 12.

Agisoft Photoscan’s point cloud is highly consistent with the laser scanner measurements; the locations and values of the deviations were the same. The average deviation from the sphere was at a similar level (3.9 mm Agisoft Photoscan; 4.3 mm C10 scanner) and the percentage of deviations exceeding 25 mm was smaller, which was the lowest of all cases and was only 0.0002%. The results from ContextCapture were much worse. The mean deviation was 7.2 mm, but 2.77% of the deviations were greater than 25 mm. Many significant deviations occurred in areas where the object had not been deformed, making it impossible to determine the actual surface deformation.

In summary, a cloud created for an object with a matte surface can give an accuracy comparable to that of laser scanning, enabling the analysis of thin-walled dome deformation. It is essential to choose software with the right computational algorithm. Due to the limited space inside a dome, it is difficult to maintain the rule of three parallel photo bases covering the same area. This, and the fact that in ContextCapture, a point cloud is created on the basis of a mesh, could have caused unidentifiable errors in surface shape.

## 7. Conclusions

The conducted research allowed for analyzing the possibility of determining the shape of a thin-walled dome surface with a few millimeters accuracy. Laser scanning provides much more stable results than the two compared measurement methods. However, they may be exposed to a decrease in accuracy caused by scattering of the beam in a thin layer of liquid on the surface of the object (rainfall or dew effect). The droplets may have different surface areas, heights, volumes, and temperatures and may cover different types of surfaces, and the light beam will fall at a different angle in each of them. Such measurements cannot be systematized because each result may be different. It is visible in the random dispersion of deviations over the entire surface of the moist object. The scattering of the laser beam may also appear in the structure of the material if the dome is made of a synthetic material. In this case, the measured distances changed approximately by a fixed value. By making an appropriate correction, taking this value into account, it was possible to determine the correct average shell-wall thickness.

A big simplification during measurements can be made through the use of rotation of the dome and taking the measurements from one station. However, measurements from the outside do not allow for obtaining a model of the upper part of the object. It may be optimal to take measurements from the inside, which give practically the same image of deformation as the measurements from the outside. 

Photogrammetric measurements, including UAV ones, allow for determining a reliable model of the surface if it is matte, which protects against the appearance of reflections. Such results were obtained for the inside of the dome. The outer side, covered with a glossy lacquer, had very large deviations in the upper, clean part of the dome. In the lower part, atmospheric pollution had accumulated, causing tarnishing of the surface, which allowed to obtain results much more similar to laser scanning.

In the case of a shell structure with a curvilinear surface shape, it is very important to use appropriate software for creating point clouds from photos. In the study, Agisoft Photoscan proved to be more effective than ContextCapture, taking into account conditions of the measurements: the limited space and restrained geometry of measurement and illumination and smooth surfaces with a small amount of characteristic points in the photos. A summary comparison of the basic parameters of all the analyzed cases is presented in Table 1.

A comparison of the measurement results with the design model may be difficult to achieve when the only homological points that can be distinguished on the dome are located at the areas of the largest deformations of the object. For a shell object with a known mathematical shape (in this case, a sphere), free fitting of the model can be applied and deviations can be determined in relation to it.

## Figures and Tables

**Table 1:** Summary comparison of the parameters of all the analyzed cases.

Sphere Model	Mean Deviation (mm)	Deviations > 25 mm (%)	Sphere Radius (m)
Assembled, outside, scanning	5.7	0.075	1.996
Mounted, outside, scanning	4.7	0.002	1.997
Mounted, outside, Agisoft Photoscan	43.9	30.313	1.966
Mounted, outside, ContextCapture	34.6	36.160	1.972
Mounted, inside, scanning	4.3	0.002	1.994
Mounted, inside, Agisoft Photoscan	3.9	0.0002	1.991
Mounted, inside, ContextCapture	7.2	2.769	1.991
